# Oxidized Albumin as a Mediator of Kidney Disease

**DOI:** 10.3390/antiox10030404

**Published:** 2021-03-08

**Authors:** Stefanny M. Figueroa, Patricio Araos, Javier Reyes, Basile Gravez, Jonatan Barrera-Chimal, Cristián A. Amador

**Affiliations:** 1 Laboratory of Renal Physiopathology, Institute of Biomedical Sciences, Universidad Autónoma de Chile, Santiago 8910060, Chile; smfigueroar@gmail.com (S.M.F.); patricio.araos@uautonoma.cl (P.A.); javier.reyes470@gmail.com (J.R.); basile.gravez@gmail.com (B.G.); 2 Instituto de Investigaciones Biomédicas, Universidad Nacional Autónoma de México, Mexico City 04510, Mexico; jbarrera@iibiomedicas.unam.mx; 3 Laboratorio de Fisiología Cardiovascular y Trasplante Renal, Unidad de Investigación UNAM-INC, Instituto Nacional de Cardiología Ignacio Chávez, Mexico City 14080, Mexico

**Keywords:** oxidized albumin, obesity, kidney disease

## Abstract

Renal diseases are a global health concern, and nearly 24% of kidney disease patients are overweight or obese. Particularly, increased body mass index has been correlated with oxidative stress and urinary albumin excretion in kidney disease patients, also contributing to increased cardiovascular risk. Albumin is the main plasma protein and is able to partially cross the glomerular filtration barrier, being reabsorbed mainly by the proximal tubule through different mechanisms. However, it has been demonstrated that albumin suffers different posttranslational modifications, including oxidation, which appears to be tightly linked to kidney damage progression and is increased in obese patients. Plasma-oxidized albumin levels correlate with a decrease in estimated glomerular filtration rate and an increase in blood urea nitrogen in patients with chronic kidney disease. Moreover, oxidized albumin in kidney disease patients is independently correlated with higher plasma levels of transforming growth factor beta (TGF-β1), tumor necrosis factor (TNF-α), and interleukin (IL)-1β and IL-6. In addition, oxidized albumin exerts a direct effect on neutrophils by augmenting the levels of neutrophil gelatinase-associated lipocalin, a well-accepted biomarker for renal damage in patients and in different experimental settings. Moreover, it has been suggested that albumin oxidation occurs at early stages of chronic kidney disease, accelerating the patient requirements for dialytic treatment during disease progression. In this review, we summarize the evidence supporting the role of overweight- and obesity-induced oxidative stress as a critical factor for the progression of renal disease and cardiovascular morbimortality through albumin oxidation.

## 1. Introduction

Obesity and a high body mass index (BMI) are related to an increased frequency of health problems [1]. This epidemic is growing and has been closely linked to kidney diseases [2], in particular, to chronic kidney disease (CKD). In this sense, a meta-analysis based on 247 cohort studies showed that overweight individuals (25 ≤ BMI < 30) had a 40% higher risk of renal disease (relative risk = 1.40; confidence interval 1.30–1.50) and that obese patients (BMI ≥ 30) exhibited even higher risk [3]. Therefore, it has been proposed that the prevention and treatment of obesity may have an important effect on the incidence and progression of renal disease, with an added benefit in preventing its substantial costs and comorbidities.

CKD is a worldwide public health problem, difficult to diagnose because it is asymptomatic in many people, affecting around 10% of the population in the majority of countries [4] and closely linked to other major diseases [5]; CKD is associated with an eightfold to tenfold augmentation in cardiovascular morbimortality and is a risk multiplier in patients with diabetes and/or hypertension [6]. CKD is defined by a sustained reduction in the estimated Glomerular Filtration Rate (eGFR, <60 mL/min per 1.73 m^2^) for three or more months, which is accompanied by structural or functional anomalies of kidneys based on urine analysis, biopsy, or imaging [7]. In addition to the reduction in eGFR, albuminuria is also considered in the risk categorization of CKD patients since it has been related to increasing cardiovascular events [8]. Indeed, the use of antihypertensive medicaments can control albuminuria and can slow CKD progression [9]. Similarly, proteinuria was transiently reduced in patients with obesity-associated renal disease under hypertensive treatment [10].

Oxidative stress is characterized by rise in reactive nitrogen species and/or reactive oxygen species (ROS) due an imbalance between excessive oxidant radicals and deficient degradation of those species by antioxidant processes. In kidney disease, excessive ROS generation favors oxidative injury, fibrosis, endothelial dysfunction, and inflammation, among other renal tissue alterations [11]. Furthermore, different antioxidants and pharmacological agents have been proposed as potential therapeutic strategies to decrease oxidative stress in adult and pediatric CKD patients [12]. Although obesity and oxidative states often coexist and facilitate the progression of albuminuric kidney disease, few publications discuss the role of posttranscriptional modification of albumin in CKD as well as the possible deleterious effects of high amounts of albumin (modified or not) on tubular epithelial cells. Moreover, since CKD is frequent in patients with diabetes mellitus, attention has been focused on albumin glycation; however, other modifications, such as albumin oxidation, have been less considered. Here, we consider not only renal handling of albumin but also the effects of albumin overload in kidney, further discussing how albumin oxidation might contribute to CKD pathogenesis and how this may particularly affect the obese patient population.

## 2. Albumin and Its Relationship with Kidney Disease

### 2.1. Albumin Structure and Its Renal Handling

**Figure 1:**
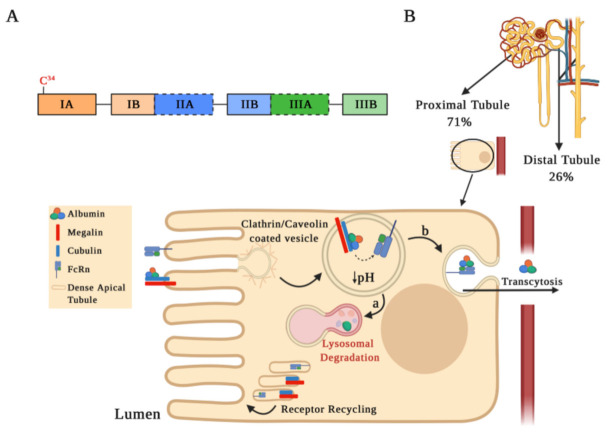
Albumin structure and its tubular reabsorption. (**A**) Schematic drawing of the human serum albumin (HSA) structure. The mature HSA presents 585 amino acids and a molecular weight of 66.5 kDa, containing three homologous α-helical domains (I, II, and III), each comprised of two sub-domains A and B. Each sub-domain is represented by a rectangular bar with a unique color. The two Sudlow’s sites, high affinity binding sites for different molecules on HSA, are marked in black dashed lines. (**B**) Albumin is partially filtered in the glomeruli and reabsorbed by receptor-mediated endocytosis into proximal (71%) and distal tubule cells (26%). This schematic representation shows albumin internalization and its degradation in the proximal tubule. Albumin binds to the megalin–cubulin complex receptor and is directed into clathrin-coated vesicles for endocytosis. Following endocytosis, endosomal acidification occurs, which causes albumin dissociation from the megalin–cubulin complex, leading albumin to bind to the neonatal Fc receptor (FcRn). Finally, albumin is transferred to the lysosomal compartment for degradation (step a) or for the transcytotic pathway (step b), while the receptors may be recycled through dense apical tubules.

Albumin is the most abundant serum protein, representing approximately 60% of the total protein content in serum. The active form of albumin corresponds to a 66 kDa globular protein of 585 amino acids organized in three domains (I, II, and III), which in turn are comprised of two sub-domains A and B (Figure 1A). Albumin is mainly synthetized in the liver and has multiple physiological functions, such as maintaining osmotic pressure, redox balance, transporting fatty acids, bilirubin, medications, hormones, vitamins, etc. [13,14,15].

Circulating albumin is filtered in small amounts at renal level (<4 mg/m^2^/h). However, three physiological situations lead to albumin presence in urine: postural, febrile, and exercise [14,16]. In all these circumstances, albuminuria is transiently elevated but absent when urine collection is performed first thing in the morning, after a feverish period, or post-recovery from strenuous exercise, respectively. Considering the above, in normal conditions, the glomerular barrier prevents the majority of albumin filtration due to its size and negative charge [16].

The glomerular filtration barrier consists of endothelial cells, a basal membrane, and podocytes, forming the main barrier that prevents filtration of plasma proteins, such as albumin into the ultrafiltrate [17]. The endothelial cells of glomerulus are covered by a negatively charged glycocalyx that reduces the filtration of anionic solutes, such as albumin [17,18,19]. In this context, studies have shown that enzymatic destruction of endothelial glycocalyx increases albuminuria, altering the glomerular pore size and load selectivity of glomerular filtration barrier [20,21]. In addition, under certain conditions, e.g., aging, diabetes, and CKD, there is a decrease in endothelial glycocalyx generating an increase in albuminuria [22,23]. Therefore, under physiological situations, molecules greater than 4 nm do not filter freely; oppositely, they are retained in serum as their size increases. However, in pathological conditions, with an increment of effective radius, different proteins are favored in crossing the filtration barrier. In this context, albuminuria may progressively increase, evidencing glomerular damage and representing an indicator of CKD prognosis [15].

Albuminuria levels are influenced by both glomerular filtration integrity barrier status and serum albumin levels. Albuminemia values between 35–50 g/L allow us to find concentrations of albumin in the renal ultrafiltrate at 22–32 mg/L [19,24]. In micropuncture studies, it has been estimated that, in the human kidney, 3.3 g of albumin are filtered daily, with an estimated tubular reabsorption of 3.2 g/day, occurring at 71% and 26% in the proximal and distal tubules, respectively [16,25]. Albumin reabsorption at the proximal tubule level is favored by endocytosis mediated by receptors located on the apical plasma membrane or by pinocytosis, through clathrin-coated vesicles or fluid-phase vesicles, respectively (Figure 1B). The most relevant receptors involved in albumin endocytosis are the neonatal Fc receptor (FcRn) and the megalin–cubilin complex [26,27]. After endocytosis, an acidification occurs in the endosomal compartment that allows for dissociation of albumin from the megalin–cubilin complex, thus promoting its binding to FcRn receptors. Albumin can be further transported into lysosomes for degradation (Figure 1B, step a) or for a transcytosis route (Figure 1B, step b), which is favored by tubular structures that mediate albumin transport to the basolateral membrane. Membrane fusion in the transcytosis route and pH increase in the peritubular capillaries promote albumin dissociation from the FcRn receptor, recycling albumin back into the apical membrane through a compartment known as “dense apical tubules” [15,26,27,28,29]. Since the binding of albumin to the FcRn receptor may be reduced by albumin modifications, such as glycosylation or carbamylation (discussed below), albumin transcytosis may not occur and albumin may enter the lysosomal pathway. This mechanism of intracellular molecular classification that leads to the preservation of physiological albumin facilitates the catabolism of chemically altered albumin [29].

Concerning the mechanisms for albumin reabsorption in distal tubules, little information is available. An in vitro model of albumin overload using MCDK cells (collecting/distal tubule cell model) suggested that albumin internalization occurs via clathrin-mediated endocytosis with the formation of early and late endosomes and that albumin is subsequently degraded in lysosomes [30].

### 2.2. Implications of Albumin Overload at Renal Level

As we mentioned before, the excessive presence of albumin in urine is considered an indicator of kidney damage [8,31], since it represents alterations in both filtration and reabsorption processes [29]. Different proteinuric nephropathies have in common increased intraglomerular pressure and increased perfusion pressure that lead to the stretching of glomerular capillaries and podocytes [32]. The excessive amount of albumin that is filtered in pathological situations and that reaches the proximal and distal tubules might trigger and amplify deleterious mechanisms that contribute to CKD progression, as described below.

Recently, with the objective of identifying the renal effects of albumin exposition, different in vivo and in vitro experimental models of albumin overload have been implemented. Albumin overload in podocytes promotes cell damage through the release of transforming growth factor beta (TGF-β) and the consequent differentiation of mesangial cells into myofibroblasts [33], with an activation of apoptotic pathways in podocytes by caspases 3/7 and the secretion of pro-inflammatory cytokines, such as tumor necrosis factor (TNF), interleukin (IL)-6, and IL-1α [34].

At the proximal tubule level, it has been determined in vitro that albumin overload stimulates the production and release of endothelin-1 (ET-1) to the basolateral compartment, accompanied by the release of chemokine (C–C motif) ligand 5 and 2 (CCL5 and CCL2, respectively) [35], both pro-inflammatory chemokines that promote leukocytes recruitment. In this sense, it has been observed that intraperitoneal albumin overload (10 mg/g/day) in mice generates renal macrophage recruitment and proteinuria after 7 days. It has been determined that these changes are accompanied by tubular dilation, glomerular remodeling. and collagen deposition in addition to an increase in IL-6, TNF-α, and TGF-β [36,37,38]. On the other hand, randomized studies performed with critically ill patients have shown that the albumin overload (“hyper-oncotic albumin”) used to stabilize hemodynamics does not generate nephrotoxic effects during the hospital stay period [39]. A study from Frenette et al. demonstrated that albumin administration in patients with cardiac surgery is associated with episodes of acute kidney damage [40]. Unfortunately, the records of prolonged follow-up in these patients are not considered or reported and the discussion concerning the albumin dose to be used in order not to induce kidney damage is still open [41]. In general, the literature indicates that albumin excess may be related to the induction of cytokines/chemokines at the renal level and with early macrophages infiltration in CKD [35,40], favoring disease progression.

Concerning the mechanisms that control protein levels in urine, it has been determined that angiotensin II (AngII) blocks the reabsorption of albumin due to a reduction in the expression of megalin in diabetic rats [42]. In particular, blockade of the renin angiotensin system may offer a reduction in proteinuria independent of blood pressure [43], allowing us to slow down the progression of CKD [44,45]. Thus, current guidelines recommend the use of AngII antagonists to control proteinuria in patients with CKD [46]. However, in advanced stages of CKD, the use of antihypertensive drugs accelerates kidney damage in adults [47,48], for which it is essential to generate new studies in these populations of patients.

## 3. Albumin Oxidation and Its Renal Pathophysiological Consequences

Albumin can undergo different posttranslational modifications that may alter its structure, hydrophobicity, net charge on its surface, redox state, drug binding capacity, and antioxidant capacity [49]. In this sense, oxidation, cysteinylation, glycation, S-nitrilation, and S-guanylation are the most relevant posttranslational albumin modifications [50].

**Figure 2:**
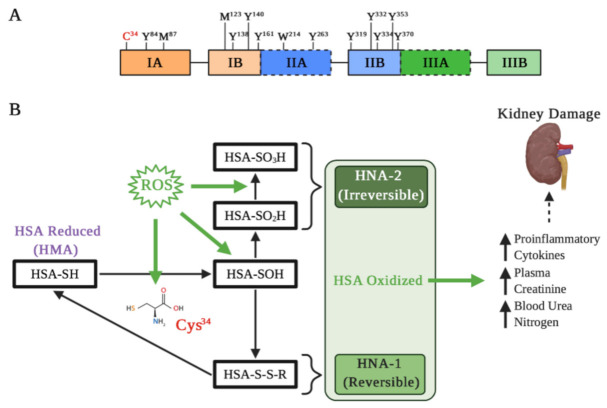
Albumin oxidation affects its properties in kidney damage. (**A**) Main residues on the active HSA sensitive to oxidation: Cys residue at position 34; Tyr residues 84, 138, 140, 161, 263, 319, 332, 334, 353, and 370; Met residues at positions 87 and 123; and Trp residue at position 214. Met-87 and Met-123 are most oxidized to methionine sulfoxide, particularly in kidney failure and diabetes. (**B**) Different types of Cys34 oxidation. Under oxidative stress induced by reactive oxygen species (ROS), albumin is oxidized and Cys34 forms a disulfide with a free cysteine or glutathione. The oxidation changes the HSA three-dimensional structure that influences binding of drugs, such as Sudlow’s site II. Depending on the extent of HSA oxidations, they can be classified as reversible (HNA-1) and irreversible (HNA-2), which may increase different pro-inflammatory cytokines and markers of kidney damage.

Albumin is constantly exposed to oxidative stress and several agents that can oxidized it [51]. These modifications of the redox state have been related to different pathological conditions at the renal level [52]. Albumin presents different amino acids susceptible to oxidation, giving itself a relevant role as an antioxidant factor in different pathologies [53]. Annibal et al. examined various chemical modifications of human serum albumin (HSA) and showed that cysteine (Cys), tryptophan, tyrosine, and methionine are the most susceptible amino acids to oxidation [54]. Particularly, Cys represents the sulfhydryl group that is essential for redox regulation of different proteins structure and function. HSA has 35 Cys residues, where 34 form disulfide bridges and with only one free sulfhydryl group in position 34 (Cys34) [55] (Figure 2A). The oxidation of albumin in Cys34 has been related to different pathologies in the liver and kidney [56,57], and particularly, albumin Cys34 represents ≈80% of the total free plasma thiols that interact with ROS and reactive nitrogen species [58].

The albumin molecule in which the Cys34 thiol group exists in a reduced form is called “human mercaptoalbumin” (HMA), while the albumin molecule in which the Cys34 thiol group exists in an oxidized state is called “human non-mercaptoalbumin” (HNA) [59,60]. The Cys34 thiol under oxidative stress undergoes its oxidation and is converted into sulfenic acid (–SOH), which is an intermediate in redox regulation by reactive species. Sulfenic acid can be converted into a disulfide (–SS–R) by binding to small groups such as Cys, homocysteine or glutathione, which allows for a reduction in the thiol group to its original form (–SH). Moreover, this form of albumin oxidation is considered reversible (HNA-1). On the other hand, if the sulfenic acid is further oxidized to sulfinic (–SO_2_H) or sulfonic acids (–SO_3_H), the resultant albumin is considered an irreversibly oxidized form (HNA-2), causing a permanent loss of its antioxidant function [53,61] (Figure 2B).

In healthy individuals, it has been reported that about 70–80% of albumin is in its HMA form, 20–30% is in a HNA-1 form, and about 5% is in a HNA-2 form [62]. However, it has been established that increasing age is positively correlated with increasing HNA, particularly the HNA-1 form [63]. A study performed on endothelial cells stimulated with oxidized albumin showed an increase in senescence together with the generation of endothelial damage due to oxidative stress and an increase in apoptosis [64]. However, it is important to note that the measurement to differentiate HNA-1 from HNA-2 is carried out by high-performance liquid chromatography, where HNA-2 and HNA-1 peaks are contiguous [65,66], which may reduce the determination accuracy.

In recent years, attention has been focused on the oxidation state of albumin in various pathologies, since an increase in oxidized proteins has been observed in patients with CKD and IgA nephropathy, among other kidney pathologies. In this sense, the plasma measurement of free albumin–Cys34 has been used as an oxidative stress marker, which is decreased in patients with IgA nephropathy [67]. In fact, the ratio between the oxidized and normal forms of HSA, represented in the redox states of Cys34, has been proposed as a marker for the systemic redox state and for estimation of the chronic disease progression [68].

As mentioned above, in people with obesity, there is an increase in oxidative stress, a stress that has also been observed in patients with CKD [69,70]. Notably, Masudo et al. positively corelated BMI with HNA [69]. Kobayashi et al. performed a multivariate analysis of a single-center cross-sectional study relating BMI, glycated albumin (GA)/glycated hemoglobin (HbA1c) ratio, and eGFR as predictors of the percent of HNA, observing a positive correlation between the real values and the predicted ones according to the equation HNA% = 24.019 + (0.204 × BMI) + (1.442 × GA/HbA1c) − (0.117 × eGFR), with a coefficient of determination R^2^ = 0.44 [71]. However, it remains to be elucidated whether these associations between obesity, oxidative stress, and oxidized albumin are part of the cause or effect of the oxidative process.

It has been shown that, in CKD patients on pre-dialysis, HNA levels are increased in relation to decreased renal function and that a positive correlation is observed between the increase in oxidized albumin (HNA-1 and HNA-2) and increased blood urea nitrogen and serum creatinine [65,69,72]. Moreover, in patients in hemodialysis, elevated levels of HNA have been reported (particularly, the HNA-1 form), with albumin being an important target in oxidative modification in these patients [63,73]. Interestingly, in patients with normal levels of albumin subjected to hemodialysis, although the albumin concentrations did not change, dialysis modified the redox state of albumin; HMA increased (from 59.68 ± 8.9% to 76.08 ± 8.4%), while HNA-1 decreased (from 38.28 ± 8.7% to 21.78 ± 8.0%), without changes in HNA-2 levels (from 2.28 ± 0.5% to 2.38 ± 0.6%) [74]. These modifications in the redox state are crucial in patients with cardiovascular disease [75], where low levels of HMA are related to a higher incidence of cardiovascular events [76].

The HNA-1 increase in renal patients is accompanied by a pro-inflammatory state, characterized by an increase in IL-6, IL-8, and IL-1β [73] (Figure 2B). In addition, it has been precisely demonstrated that HNA-1, unlike HNA-2, has the ability to evoke a pro-inflammatory response in human leukocytes, causing an increase in IL-1β, IL-6, TNF-α, and IL-1α [77]. In this same line, it has been demonstrated that neutrophils stimulated with albumin obtained from patients with high levels of HNA-1 and HNA-2 show an increase in gene expression related to ROS production, pro-inflammatory cytokines, and high levels of neutrophil gelatinase-associated lipocalin [78], a glycoprotein which is considered a biomarker for acute and CKD [79,80] and for cardiovascular disease [81,82]. Similarly, it has been demonstrated in experimental proteinuric animals that the blood pressure increase positively correlates with oxidized albumin in serum [83], which also is suggested in patients in peritoneal dialysis [84]. In general, the pro-inflammatory state is crucial for development and progression of different kidney diseases; thus, evaluating not only albumin levels but also its redox state could be crucial in the prognosis of kidney disease.

## 4. Other Modifications of Albumin

### 4.1. Glycated Albumin and the Progression of Diabetic Kidney Disease

Weight gain and increased BMI are major contributors to the mounting prevalence of diabetes. Moreover, diabetes is a main cause of CKD, namely diabetic kidney disease (DKD). The persistent hyperglycemic environment during diabetes favors nonenzymatic albumin glycation (Maillard reaction), generating Amidori GA [85]. This reaction can occur between free Lys and Arg or the free thiol group of albumin-Cys34, and glucose or fructose. Twenty nine sites of glycation in HSA have been observed, involving 18 Lys residues [86]. During diabetes, GA plasma levels increase by 2- to 3-fold, and plasma GA has been proposed as a useful biochemical marker to monitor glucose control in diabetic patients [85]. In this sense, high GA levels are shown to be predictive of major adverse cardiac events in type 2 diabetic patients as well as of atherosclerosis and coronary artery disease [87,88,89,90]. Likewise, high GA levels are associated with augmented arterial stiffness in nondiabetic persons [91].

Elevated GA levels have been associated with kidney disease progression, augmented mortality rates, and increased risk for cardiovascular adverse events. In patients with treated type 2 diabetes (HbA1c < 7.2%), GA variability was independent from DKD onset and progression, determined as a composite renal outcome of the progression rates of CKD, albuminuria, and renal death [92]. In a cohort of 444 diabetic dialysis patients, GA predicted the risk of death and hospitalization. Moreover, a 5% increase in GA has been related to a higher risk for mortality [93]. In a cohort of 176 hemodialysis kidney failure patients with a median follow-up of 51 months, lower GA levels predicted better long-term cumulative survival [94]. Similarly, high GA levels predicted mortality in diabetic hemodialysis patients [95]. In agreement with these findings, augmented cardiovascular morbidity and shortened survival were found in diabetic patients with end stage renal disease and high GA levels [96]. The strong association of high GA levels with kidney disease progression and adverse cardiovascular effects suggests that GA exerts a pathological role and contributes to CKD progression. This idea is supported by observations showing that the infusion of glycated proteins induces features of DKD in rodents [97,98]. Moreover, it has been demonstrated that GA enhances the glomerular basement membrane permeability, thus contributing directly to albuminuria and renal injury [99].

In vitro studies have delineated possible mechanisms by which GA might contribute to kidney fibrogenesis. Mouse mesangial cells cultured in normal glucose and then exposed to GA displayed upregulation in TGF-β and TGF-β receptor type II mRNA levels compared to the group exposed to non-glycated albumin, thus showing a direct profibrotic effect of GA [100]. Interestingly, since the half-life of GA is around 2 weeks, it may continue to influence the TGF-β pathway even in normoglycemia [100]. Mouse mesangial cells incubated with glycated serum proteins increase collagen IV mRNA levels, one of the main components of the expanded mesangial matrix in DKD, whereas neutralization of GA prevented this effect, thus pointing out the specific role of GA in collagen IV induction in mesangial cells [101]. These collagen IV increases and TGF-β production are likely mediated by protein kinase C (PKC) activation, since pharmacological inhibition of PKC and PKC-β activity blunts these inductions [102,103]. Indeed, PKC activation triggered by GA has been documented in glomerular cells [103]. Likewise, GA increased PKC and PKC-β1 isoform activity in rat and mouse mesangial cells as well as in rat glomerular endothelial cells in which GA increased PKC activity despite normal glucose concentrations in the medium [102]. Furthermore, glomerular endothelial cells showed increased expression of fibronectin and type IV collagen in response to GA stimulation [101].

The in vivo relevance of these previous observations has been confirmed in diabetic mice. The chronic administration of antigen-binding fragments against GA (A717) decreased plasma GA concentration and strongly reduced albuminuria, glomerular pathology, and renal dysfunction, providing evidence of a role for GA not only as a biomarker but also as a mediator of kidney injury in DKD [104,105]. Further studies have demonstrated that GA induces TGF-β production, oxidative stress, and NFκB activation in macrophages, which might have also an impact on the pathophysiology of DKD [106]. In addition, Neelofar et al. found that GA develops structural alterations that lead to the creation of neo-epitopes not present in the native molecule, being highly immunogenic; indeed, autoantibodies against GA were detected in CKD and non-CKD diabetic patients [107].

Altogether, the evidence points out a deleterious role of GA in kidney disease by stimulating the production of fibrotic mediators that may alter kidney structure favoring albuminuria and kidney dysfunction. Moreover, GA associates with adverse cardiovascular and atherosclerotic effects that could also be mediated by GA effects on macrophages and the inflammation processes. However, it is necessary to further explore GA effects on kidney structure and how it has a pathological role not only as a biomarker of glucose control.

### 4.2. Albumin Cysteinylation or S-Thiolation

HSA undergoes cysteinylation or S-thiolation, or the addition of another cysteine to Cys34 via a disulfide bond [108]. In healthy young persons, ≈70% and ≈30% of HSA presents Cys34 as a free sulfhydryl group and as a reversible mixed disulfide with other thiols of low molecular weight, respectively [109]. On the other hand, Brioschi et al. found that S-thiolation of HSA was augmented in the plasma of heart failure patients, particularly in high severity cases (class III and class IV according to the New York Heart Association classification) [110]. In addition, the authors found that S-thiolated HSA correlated with deficient antioxidant activity in plasma, suggesting that S-thiolation promoted changes in HSA at the structure and function level [110]. Similar results were found in eight end-stage renal disease patients with at least 3 months of hemodialysis, observing an increase in pre-dialysis S-thiolation levels of HSA compared to control patients. Interestingly, the post-dialysis patients showed a reduction in HSA S-thiolation [111]. Nakashima et al. showed that S-thiolation exists not only at the single free thiol group but also at multiple cysteine residues in the disulfide bonds of HSA [55]. Moreover, dynamic thiol/disulfide homeostasis has been implicated in other disorders. In this sense, a case–control study of obese and healthy children aged between 4 and 18 years showed that obese patients presented lower native thiol (SH) and total thiol (SH+) concentrations than controls. However, the authors evaluated these parameters in total plasma proteins and not specifically in HSA [112]. Therefore, more studies are necessary to understand the relevance of S-thiolation in the HSA function.

### 4.3. Albumin S-Nitrosylation and S-Guanylation

Cysteine residues on HSA can also experiment with S-nitrosylation or nitric oxide (NO) addition due to the reactivity of sulfhydryl groups [113]. Apparently, HSA S-nitrosylation can reduce the uncoupling of endothelial NO synthase, improving systolic and diastolic function, and myocardial perfusion in a model of ischemia/reperfusion in pigs [114]. In this sense, this modification has been proposed as a potential NO donor with therapeutic applications [115].

Posttranslational modifications of HSA involve S-guanylation and dehydroalanine conversion. S-guanylation of Cys34 is a change in HSA that occurs when an 8-nitroguanosine 3′,5′-cyclic monophosphate group reacts with sulfhydryl residues [116]. The available evidence indicates that this modification may decrease the HSA ability to bind drugs, conferring some antimicrobial capacity [116]. However, albumin S-nitrosylation and S-guanylation have not yet been related to CKD in obese patients.

## 5. Conclusions

**Figure 3:**
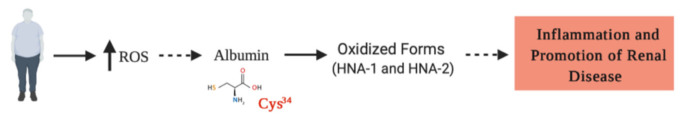
Reactive oxygen species (ROS) are increased in obese people, favoring the oxidized form of HSA (HNA), which in turns, generates an increase in proinflammatory cytokines. Here, we hypothesized (discontinuous lines) that the HNA, particularly oxidized at Cys34, may be generated as a result of obesity, promoting the progression of inflammatory and degenerative injuries, which are crucial for the progression of chronic kidney disease.

Although there is not enough data showing a difference between HNA-1 and HNA-2 for CKD risk in obese patients, the literature available suggests that albumin oxidation reflects a state of pro-inflammatory renal injury that may favor CKD progression (Figure 3), in particular, considering that aging patients, a population that present a higher risk for CKD, also present higher HNA-1 levels. Furthermore, albumin overload in the tubular compartment is associated with increased inflammatory mediators, which may also amplify tissue injury in CKD.

We believe that new studies analyzing the HNA forms in obese patients before and after renal transplant/hemodialysis will provide crucial evidence supporting the relation between oxidized albumin and renal health. In addition, in vitro experiments performed on podocytes, and mesangial and tubular cells challenged with HNA-1 and HNA-2 will provide new possible mechanisms by which oxidized albumin promotes kidney damage.
